# Case Report: Three-fraction lattice radiotherapy followed by VMAT concurrent chemoradiotherapy in locally advanced non–small cell lung cancer

**DOI:** 10.3389/fonc.2026.1740098

**Published:** 2026-01-28

**Authors:** Chunyang Zhou, Hong Liu, Chao Yan, Tao Yang, Aijie Yang, Xiangyong Liu, Xiaoli Liu, Zijian Wang

**Affiliations:** 1 Department of Radiotherapy, Qilu Hospital of Shandong University, Shandong University, Qingdao, Shandong, China; 2 Qilu Hospital of Shandong University, Shandong University, Qingdao, Shandong, China

**Keywords:** adaptive replanning, lattice radiotherapy, non-small cell lung cancer, spatially fractionated radiotherapy, volumetric modulated arc therapy

## Abstract

**Background:**

In unresectable stage III non–small cell lung cancer (NSCLC) with bulky primary tumors and extensive nodal irradiation volumes, dose escalation during concurrent chemoradiotherapy (cCRT) is often limited by the tolerance of organs at risk (OARs). Lattice radiotherapy (LRT), a form of spatially fractionated radiotherapy (SFRT), generates a characteristic intratumoral peak–valley dose distribution, which may enable focal dose intensification without increasing the dose to surrounding normal tissues.

**Case description:**

A 65-year-old man with unresectable stage IIIC (cT4N3M0) poorly differentiated squamous cell carcinoma of the right upper lobe first underwent 3 fractions of LRT. He then received volumetric modulated arc therapy (VMAT) delivered concurrently with platinum-doublet chemotherapy. During treatment, cone-beam CT (CBCT) demonstrated marked tumor regression; a second CT simulation was performed and target volumes were recontoured, reducing the primary gross tumor volume (GTVp). The patient completed the remaining 14 fractions using the new plan. Treatment was well tolerated, with only grade 1 sore throat and reversible anemia, both improving with supportive care. One month after completion, chest CT showed substantial tumor shrinkage consistent with a partial response (PR) per RECIST v1.1. Approximately 1.5 months post-treatment, radiation-induced pneumonitis occurred and improved with anti-infective/supportive management. The Eastern Cooperative Oncology Group (ECOG) performance status remained 0–1 during follow-up.

**Conclusions:**

This case suggests that, in unresectable stage III pulmonary squamous cell carcinoma, a combined strategy of limited-fraction LRT plus VMAT with concurrent chemotherapy, together with on-treatment adaptive replanning, can achieve favorable short-term efficacy and acceptable toxicity while maintaining OAR doses within constraints. This approach provides a practical reference for individualized radiotherapy with complex target volumes.

## Introduction

Non–small cell lung cancer (NSCLC) accounts for over 80% of all lung cancers, and a substantial proportion of patients present at diagnosis with unresectable, locally advanced disease. For these patients, the standard of care is concurrent chemoradiotherapy (cCRT) followed by programmed death-ligand 1 (PD-L1) immunotherapy (1). Radiotherapy is typically delivered with conventional fractionation to a total dose of approximately 60–66 Gy (2 Gy/fraction over ~6–6.5 weeks), aiming to maximize tumor control while respecting organs at risk (OARs) dose constraints. However, when the primary tumor is bulky and treatment must encompass extensive nodal irradiation volumes, achieving a uniform prescription dose is constrained by normal-tissue tolerances; meaningful dose escalation becomes difficult, and the addition of systemic chemotherapy further increases the toxicity burden (2).

Spatially fractionated radiotherapy (SFRT) encompasses two-dimensional GRID and its three-dimensional evolution, lattice radiotherapy (LRT). The central concept is to create an intratumoral peak–valley dose distribution, allowing very high doses to be concentrated within selected tumor regions (“vertices”) while maintaining the dose to surrounding normal tissues within constraints. In LRT, multiple spherical high-dose vertices are placed entirely within the tumor volume to achieve focal dose intensification without increasing the dose to surrounding normal structures (3).

Here, we report a patient with unresectable stage IIIC (cT4N3M0) locally advanced pulmonary squamous cell carcinoma treated with 3 fractions of LRT followed by conventionally fractionated volumetric modulated arc therapy (VMAT) delivered concurrently with platinum-doublet chemotherapy (paclitaxel plus carboplatin). The regimen achieved favorable short-term efficacy with acceptable safety and may provide a practical technical reference for similar patients.

## Case presentation

**Figure 1:**
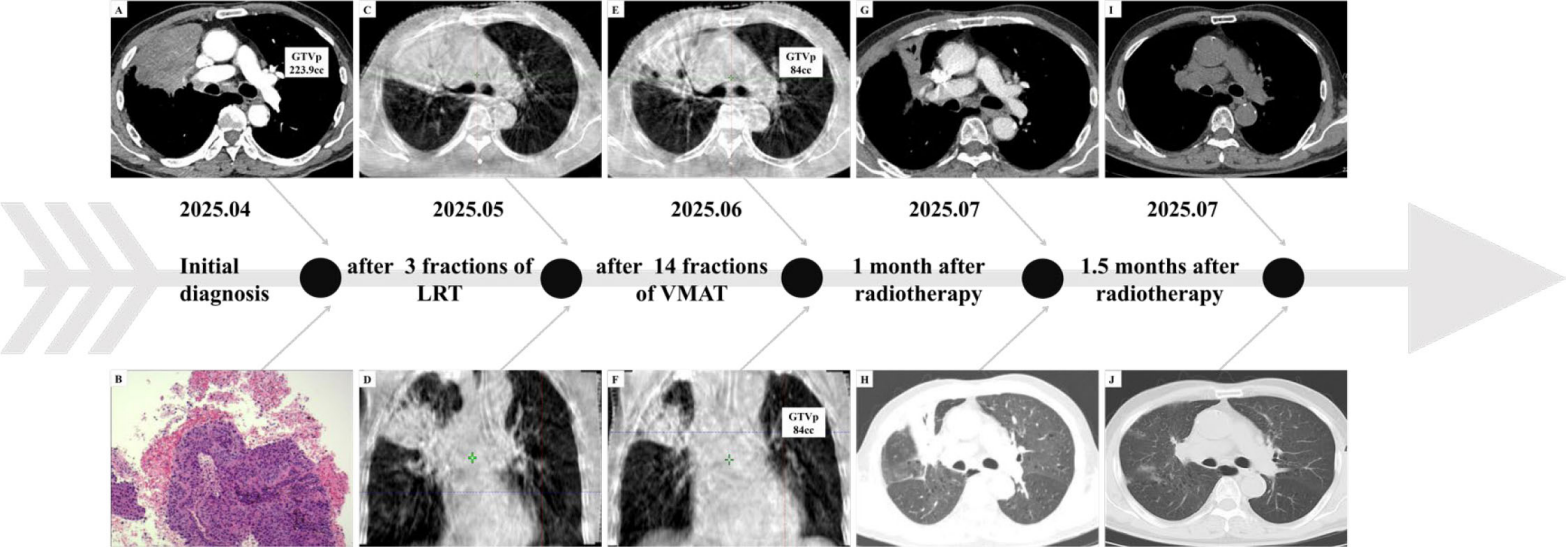
Timeline of treatments. **(A)** The initial chest CT detected a pulmonary tumor. **(B)** Pathology of the right upper-lobe tumor. **(C, D)** On-treatment CBCT after 3 fractions of LRT. **(E, F)** On-treatment CBCT after 17 fractions (3 LRT + 14 VMAT). **(G, H)** Chest CT 1 month after completion of 31 fractions of radiotherapy. **(I, J)** Chest CT 1.5 months after radiotherapy at follow-up.

A 65-year-old man, a never-smoker with a 2-year history of hypertension and no family history of malignancy, was incidentally found to have a right lung mass during hospitalization for hemorrhoid treatment. He reported intermittent irritative cough with scant white sputum, without hemoptysis, chest pain, fever, or chills. Chest computed tomography (CT) demonstrated a large apical mass in the right upper lobe (axial dimensions approximately 98 × 79 mm) with multiple enlarged mediastinal lymph nodes; cervical ultrasound showed multiple lymph nodes with preserved architecture. CT-guided percutaneous lung biopsy confirmed poorly differentiated squamous cell carcinoma (**Figure 1A**). Programmed death-ligand 1 (PD-L1, SP263) immunohistochemistry showed a tumor proportion score (TPS) of ~90% (**Figure 1B**). In the absence of bronchoscopy or invasive mediastinal staging procedures, nodal assessment relied on radiographic findings. Baseline staging utilized contrast-enhanced CT of the neck, chest, abdomen, and pelvis, alongside contrast-enhanced brain MRI; PET/CT was not performed. Following multidisciplinary evaluation, nodal metastasis was suspected in stations 2R, 2L, 3A, 4R, 6, 7, and 10R based on CT features, leading to an N3 classification. Consequently, the disease was staged as cT4N3M0 (Stage IIIC, 8th edition TNM) and deemed unresectable. In accordance with National Comprehensive Cancer Network (NCCN) guidelines, the treatment plan comprised 3 fractions of LRT followed by conventionally fractionated VMAT. This was delivered concurrently with a weekly platinum-doublet chemotherapy regimen consisting of albumin-bound paclitaxel (200 mg) and carboplatin (180 mg). Alternative initial strategies were excluded.

**Figure 2:**
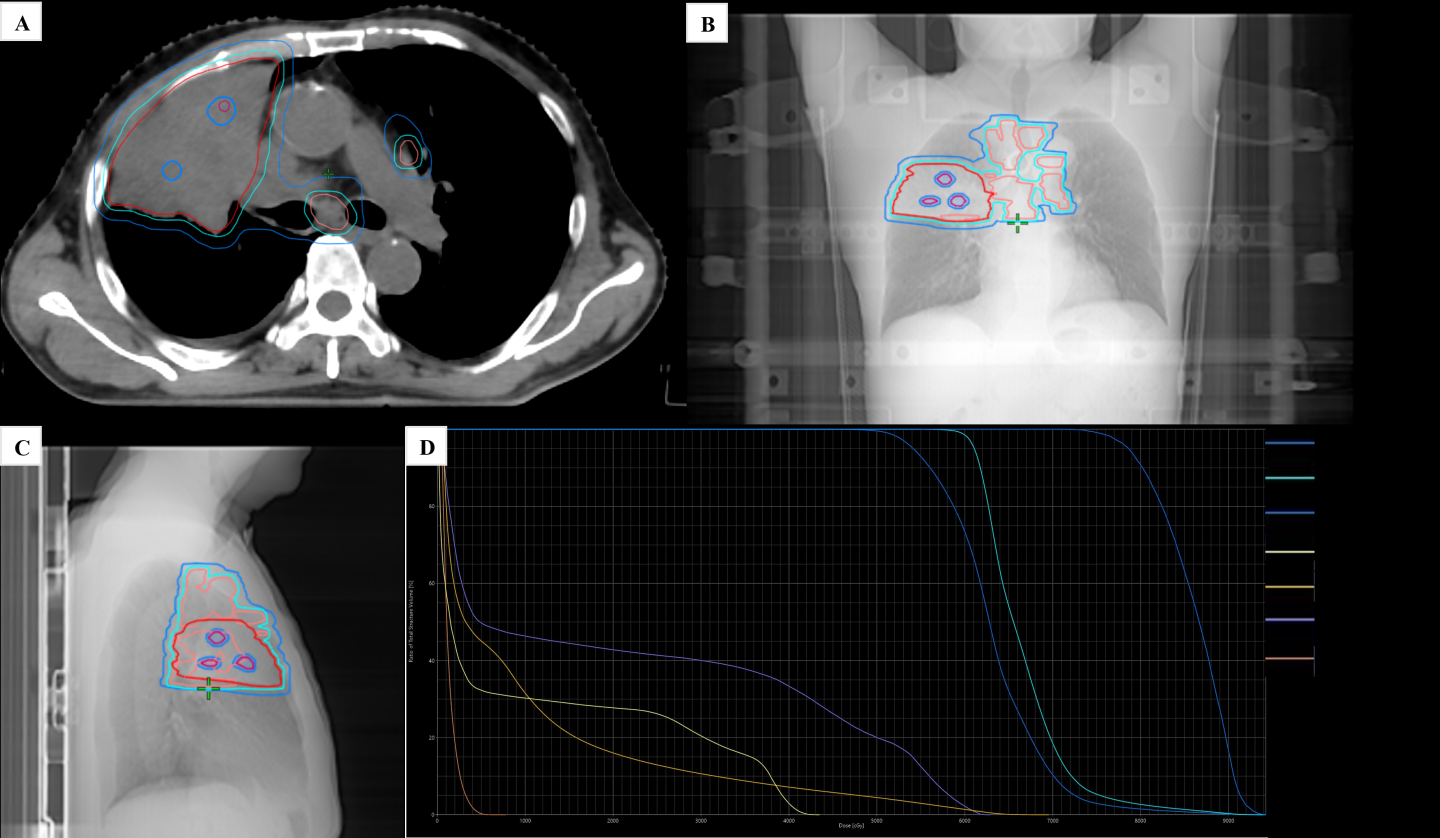
Dose distribution and Dose–volume histogram (DVH) for target volumes. **(A)** Axial plane. **(B)** Coronal plane. **(C)** Sagittal plane. **(D)** DVH chart. In **(A–C)**, the purple contour represents GTVv, the red contour represents GTVp, the pink contour represents GTVn, the cyan contour represents PTV60, and the blue contour represents PTV50.

Given the apical location in the right upper lobe and minimal respiratory excursion, conventional three-dimensional CT simulation with a thermoplastic body mask immobilization was used. The primary gross tumor volume (GTVp) was contoured as the large right-upper-lobe mass with a volume of 223.9 cc. LRT planning parameters were as follows: To limit dose to surrounding normal tissues, GTVp was isotropically contracted by 1 cm to define the lattice volume (VL) for vertex placement. Within VL, three spherical high-dose vertex volumes (GTVv) were placed in a relatively uniform arrangement, while deliberately avoiding major intratumoral bronchovascular structures (**Figure 2**). Simulation was performed using a non-contrast planning CT. The planning CT was co-registered with the prior contrast-enhanced diagnostic CT, which was used as a visual reference during contouring and vertex placement; vertices were adjusted to avoid intratumoral structures demonstrating vascular enhancement.

**Figure 3:**
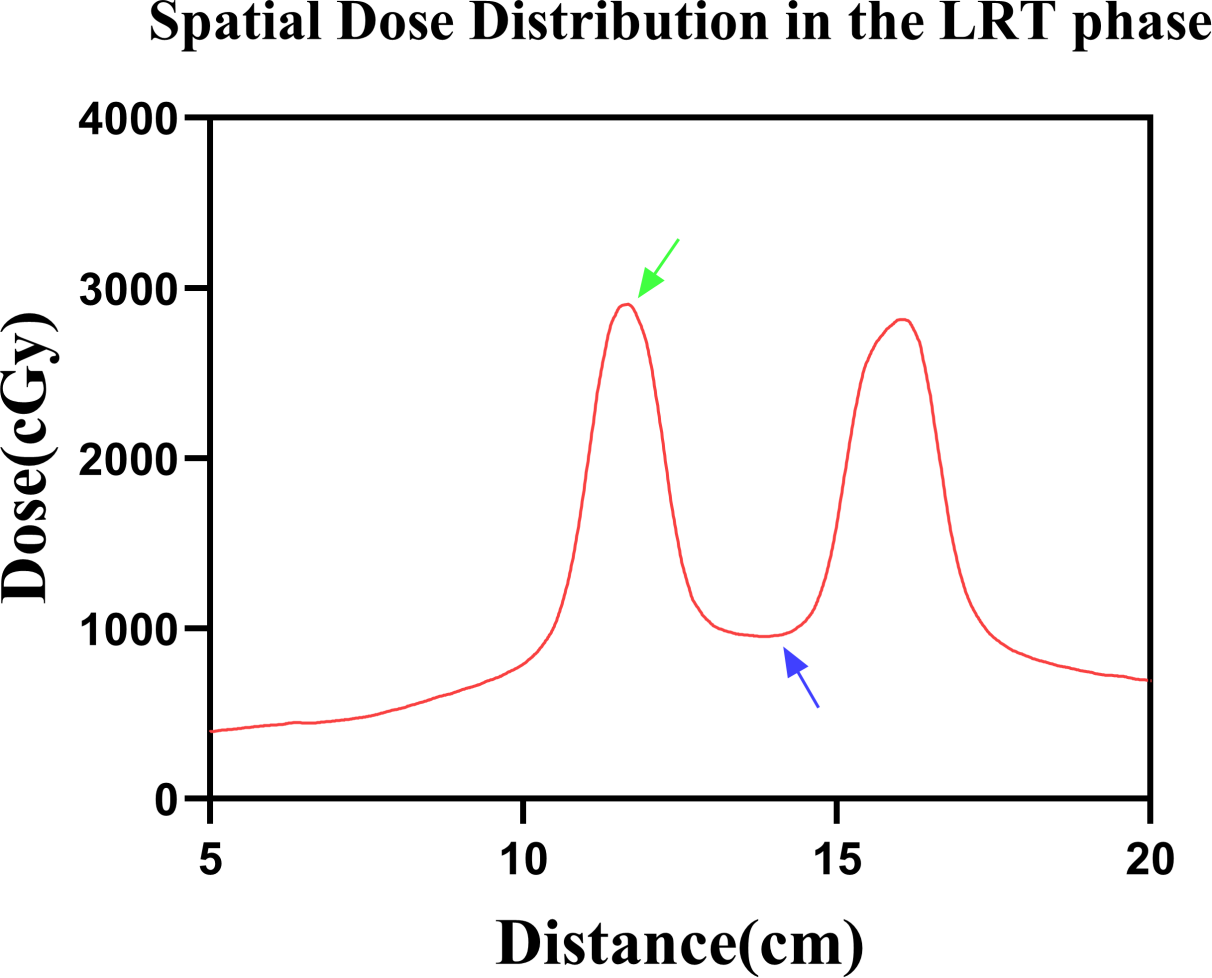
Spatial dose distribution in the LRT phase. The green arrow indicates the peak-dose region, and the blue arrow indicates the valley-dose region.

In accordance with consensus guidance, vertex geometry and dosimetry were documented as follows: vertex diameters were 1.6 cm, 1.5 cm, and 1.5 cm; center-to-center spacings were 4.3 cm, 4.1 cm, and 3.9 cm; the combined vertex volume was 2.3 cm³ (≈1.03% of GTVp). GTVv was expanded by 0.3 cm to generate the planning target volume for vertices (PTVv). The prescription to PTVv was 24 Gy in 3 fractions (8 Gy/fraction; biologically effective dose for α/β = 10, BED_10_ ≈ 43.2 Gy). The vertex-center peak dose was 29.07 Gy, valley dose was 9.53 Gy, yielding a peak–valley dose ratio (PVDR) of 3.05 (**Figure 3**).

Upon transition to conventionally fractionated VMAT, target definitions and prescriptions were as follows (**Figure 2**). The primary GTVp and involved nodal GTVn were expanded with a 3D isotropic margin of 6 mm to generate the high-dose planning target volume (PTV60). CTV50 was individualized for this patient with N3 disease: it included a 6-mm expansion around the primary tumor volume (CTVp) together with the nodal regions corresponding to the involved/suspected stations (2R, 2L, 3A, 4R, 6, 7, and 10R). CTV50 was subsequently expanded by 5 mm to generate PTV50. No elective/prophylactic irradiation of radiographically uninvolved nodal stations was performed. Prescriptions were: PTV60, 59.36 Gy in 28 fractions (2.12 Gy/fraction over ~6 weeks; BED_10_ ≈ 71.9 Gy); and PTV50, 50.4 Gy in 28 fractions (1.8 Gy/fraction; BED_10_ ≈ 59.5 Gy). Planning objectives required ≥95% of PTV60 to receive 100% of the prescription dose, with ≤2% of PTV60 receiving >107% of prescription; analogous homogeneity criteria were applied to PTV50.

OAR dose constraints were set as follows: spinal cord Dmax < 45 Gy; esophagus Dmean < 27 Gy and Dmax < 62 Gy; heart Dmean < 25 Gy (V30 < 40%, V40 < 30%); and bilateral lungs (GTV-excluded) Dmean < 15 Gy (V20 < 28%, V5 < 60%). The finalized DVH was generally consistent with these constraints; key values were: esophagus Dmean = 22.02 Gy and Dmax = 62 Gy; heart Dmean = 1.37 Gy and V5 = 0.29%; and bilateral lungs (GTV-excluded) Dmean = 10.36 Gy, V5 = 45%, V20 = 16%, V30 = 11%. VMAT was delivered on a Varian linear accelerator with daily cone-beam CT (CBCT) for setup verification.

**Figure 4:**
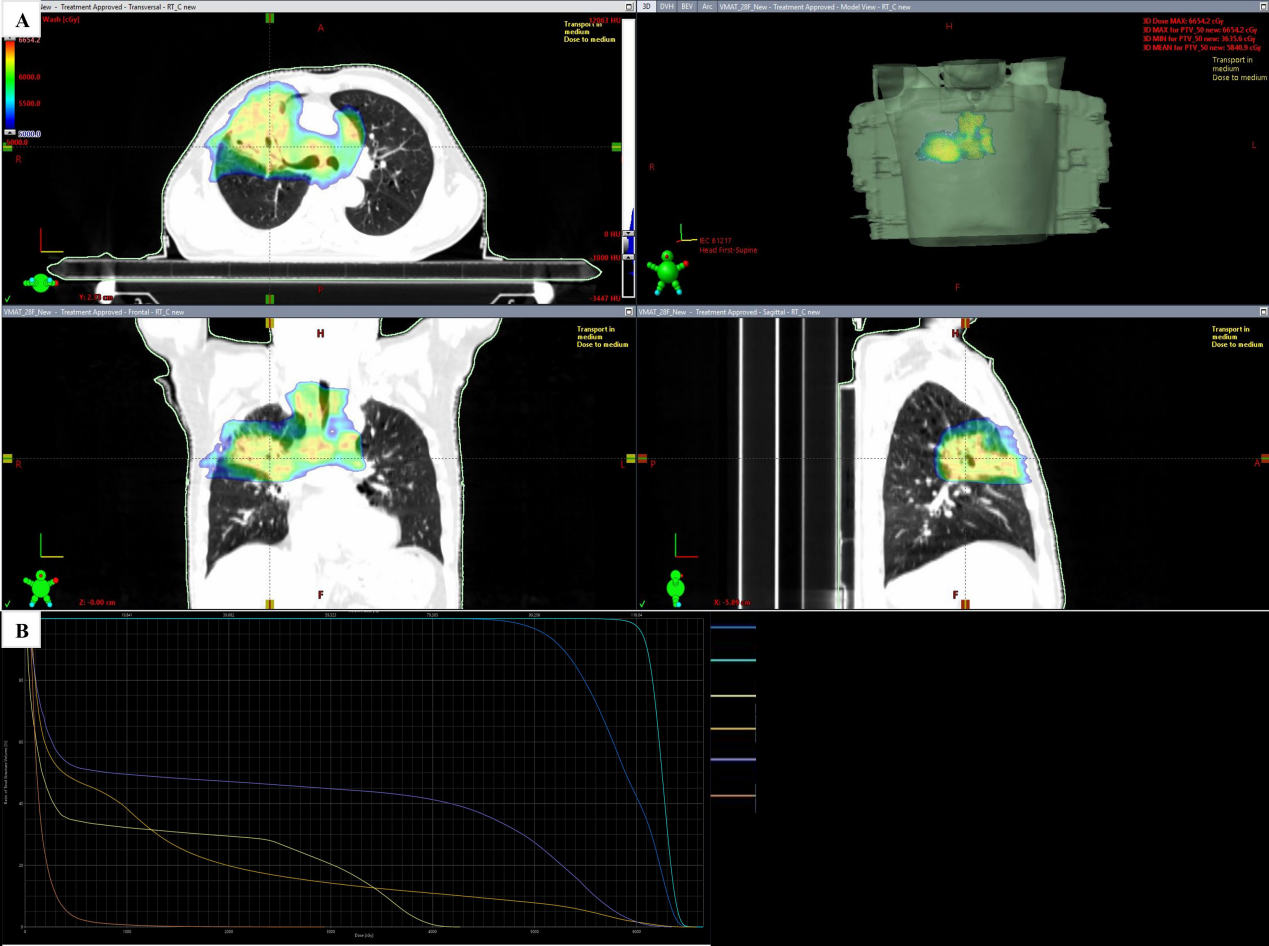
Dose distribution and DVH for replanned target volumes. **(A)** Target-volume dose distribution with hotspots. **(B)** DVH chart.

The patient completed 3 fractions of LRT (**Figures 1C, D**) followed by 14 fractions of conventionally fractionated VMAT together with weekly concurrent chemotherapy. On-treatment CBCT demonstrated marked tumor regression, with GTVp decreasing from 223.9 cc (**Figure 1A**) to 84 cc (**Figures 1E, F**). After fraction 17, a second CT simulation was performed and mid-course replanning was implemented: the primary gross tumor volume (GTVp) was recontoured to its reduced extent while keeping OAR dose constraints unchanged, and the remaining 14 fractions were delivered using the new plan (**Figure 4**).

Beginning around fraction 22, the patient developed mild sore throat, graded as Common Terminology Criteria for Adverse Events (CTCAE) v5.0 grade 1, which improved with supportive mucosal care. Hematologic evaluation showed mild anemia that improved after iron supplementation and hematopoietic therapy; in view of his history of hemorrhoids, chronic blood loss was considered contributory. At the first post-treatment follow-up (approximately 1 month after completion), the patient reported no cough, sputum production, hemoptysis, or dyspnea; chest CT showed a substantial reduction in tumor size (**Figures 1G, H**). Per RECIST v1.1, the response was partial response (PR). At ~1.5 months after completion, the patient developed mild cough, and chest CT revealed radiation field–concordant pneumonitis (**Figures 1I, J**). Symptoms improved following 2 weeks of symptomatic antitussive therapy. Given the patient’s stable pulmonary status, consolidation durvalumab was initiated at 700 mg q2w. At a telephone follow-up on 30 December 2025, the patient reported that he remained clinically well with excellent stamina and without any new or worsening symptoms (including cough), and no recurrence had been reported to date. By telephone report, he described activity consistent with a Karnofsky Performance Status (KPS) of 100 and ECOG 0, and his body weight had increased by approximately 10 kg compared with the start of treatment. He had received approximately 5 months of durvalumab (≈ 10 cycles; 700 mg q2w) as of 30 December 2025, and treatment was ongoing.

## Discussion

For patients with stage III locally advanced NSCLC characterized by bulky primary tumors and bilateral, multistation mediastinal nodal involvement, conventional uniform-dose cCRT carries a substantial risk of radiation pneumonitis and esophagitis; attempts to intensify systemic chemotherapy to enhance cytoreduction can further increase toxicity, compromise quality of life, and jeopardize completion of therapy. Stage III NSCLC is highly heterogeneous, and no single regimen is universally optimal; individualized strategies remain necessary (4). Despite advances in precision medicine and multimodality therapy, long-term outcomes remain modest—particularly in stage IIIC—underscoring the need for approaches that improve locoregional control while maintaining safety.

The central aim of radiotherapy is to deliver a tumor-controlling dose to the target while minimizing exposure of OARs. Historically, concerns about excessive skin dose with early low-energy X-rays prompted exploration of spatially fractionated delivery; early “GRID” techniques produced spatially segmented dose patterns and are considered among the first clinical applications of SFRT (3). With the advent of megavoltage LINACs, GRID waned clinically; however, renewed LINAC-based GRID experience suggested distinctive normal-tissue sparing and encouraging efficacy in bulky tumors (5, 6), rekindling interest in SFRT.

Unlike conventional radiotherapy, which prioritizes dose uniformity, SFRT intentionally delivers a highly heterogeneous spatial distribution by placing high-dose volumes (e.g., grid- or lattice-like “peaks”) within the tumor in one or a few fractions, while the remainder of the tumor and adjacent normal tissues receive lower “valley” doses. The therapeutic concept relies on spatial (rather than purely temporal) fractionation: peaks produce direct cytotoxicity in selected tumor volumes, whereas valleys mitigate normal-tissue injury and may contribute to overall efficacy through non-targeted biological effects (7). Proposed biological mechanisms can be grouped into four domains: vascular effects; cell migration/trafficking; bystander and abscopal signaling; and immune modulation (8–10).

SFRT is commonly implemented in four forms: two-dimensional GRID, three-dimensional LRT, minibeam radiotherapy (MBRT), and microbeam radiotherapy (MRT). GRID delivers a single, spatially segmented high dose in 2D (11); LRT extends GRID into 3D by placing multiple spherical high-dose “vertices” within the tumor, enabling more rational intratumoral dose sculpting while limiting dose to adjacent normal tissues (3, 12). Against this background, practical planning choices—vertex size/spacing, inward lattice margin, and dose/fractionation—become pivotal for clinical translation in lung cancer.

Building on these principles, in this stage IIIC, bulky locally advanced NSCLC case, three-fraction LRT followed by conventionally fractionated VMAT delivered concurrently with platinum-doublet chemotherapy produced rapid volumetric regression with acceptable acute toxicity. Moreover, advances in LINACs and image-guided radiotherapy have facilitated the safe delivery of LRT in the thorax.

In this case, we used 1.5–1.6 cm vertex diameters with ~4 cm center-to-center spacing and defined a 1 cm inward lattice margin (VL) to keep vertices intratumoral and preserve valley sparing—choices that align with practical recommendations for LRT planning (typical 0.5–1.5 cm vertex diameters and 2–5 cm spacing, with a 1–2 cm inward margin) (13). Prior studies have reported vertex doses ranging from 2.4 to 20.0 Gy per fraction, delivered in 1–5 fractions (13, 14). Within this framework—and recognizing that vertex dose and fractionation must be individualized to anatomy, motion, and OAR proximity—we selected 24 Gy in 3 fractions to PTVv (BED_10_ ≈ 43.2 Gy) to create intratumoral peaks while respecting OAR constraints and enabling a seamless transition to conventionally fractionated VMAT-based cCRT in our case.

Taken together, combining LRT-enabled spatial dose escalation with VMAT-based cCRT may offer a pragmatic path to improve early tumor shrinkage and short-term outcomes in selected patients with bulky, complex targets while maintaining OAR safety. Prospective work is needed to clarify: 1. Optimal combinations of vertex geometry (diameter, spacing, number) and dosimetric metrics (e.g., PVDR); 2. Differential benefit across histologic subtypes, tumor volumes, and anatomical sites, and the sources of response heterogeneity; 3. The safest and most effective sequencing of LRT with conventional radiotherapy and systemic therapy within multimodal regimens.

## Conclusions

In this unresectable stage IIIC pulmonary squamous cell carcinoma, a combined strategy of 3 fractions of LRT dose intensification followed by conventionally fractionated VMAT-based cCRT with mid-course replanning achieved marked early tumor shrinkage with acceptable acute toxicity. Best response at first follow-up was PR by RECIST v1.1. Our experience supports that, under controlled OAR exposure, LRT can deliver intratumoral dose “peaks” for bulky disease; when integrated with VMAT and guideline-concordant systemic therapy, this approach may enhance locoregional control, modulate the tumor immune microenvironment, and create favorable conditions for subsequent consolidation immunotherapy. Prospective studies are warranted to refine lattice geometry, dosimetry, etc.

## Data Availability

The original contributions presented in the study are included in the article/supplementary material. Further inquiries can be directed to the corresponding author.

## References

[1] Galluzzi L Aryankalayil MJ Coleman CN Formenti SC . Emerging evidence for adapting radiotherapy to immunotherapy. Nat Rev Clin Oncol. (2023) 20:543–57. doi: 10.1038/s41571-023-00782-x, PMID: 37280366

[2] Offin M Shaverdian N Rimner A Lobaugh S Shepherd AF Simone CB 2nd . Clinical outcomes, local-regional control and the role for metastasis-directed therapies in stage iii non-small cell lung cancers treated with chemoradiation and durvalumab. Radiother Oncol. (2020) 149:205–11. doi: 10.1016/j.radonc.2020.04.047, PMID: 32361014 PMC8239428

[3] Li H Mayr NA Griffin RJ Zhang H Pokhrel D Grams M . Overview and recommendations for prospective multi-institutional spatially fractionated radiation therapy clinical trials. Int J Radiat Oncol Biol Phys. (2024) 119:737–49. doi: 10.1016/j.ijrobp.2023.12.013, PMID: 38110104 PMC11162930

[4] Miller M Hanna N . Advances in systemic therapy for non-small cell lung cancer. BMJ. (2021) 375:n2363. doi: 10.1136/bmj.n2363, PMID: 34753715

[5] Buckey C Stathakis S Cashon K Gutierrez A Esquivel C Shi C . Evaluation of a commercially-available block for spatially fractionated radiation therapy. J Appl Clin Med Phys. (2010) 11:3163. doi: 10.1120/jacmp.v11i3.3163, PMID: 20717082 PMC5720442

[6] Stathakis S Esquivel C Gutierrez AN Shi C Papanikolaou N . Dosimetric evaluation of multi-pattern spatially fractionated radiation therapy using a multi-leaf collimator and collapsed cone convolution superposition dose calculation algorithm. Appl Radiat Isot. (2009) 67:1939–44. doi: 10.1016/j.apradiso.2009.06.012, PMID: 19632125

[7] Duriseti S Kavanaugh J Goddu S Price A Knutson N Reynoso F . Spatially fractionated stereotactic body radiation therapy (Lattice) for large tumors. Adv Radiat Oncol. (2021) 6:100639. doi: 10.1016/j.adro.2020.100639, PMID: 34195486 PMC8233471

[8] Jagodinsky JC Vera JM Jin WJ Shea AG Clark PA Sriramaneni RN . Intratumoral radiation dose heterogeneity augments antitumor immunity in mice and primes responses to checkpoint blockade. Sci Transl Med. (2024) 16:eadk0642. doi: 10.1126/scitranslmed.adk0642, PMID: 39292804 PMC11522033

[9] Fernandez-Palomo C Chang S Prezado Y . Should peak dose be used to prescribe spatially fractionated radiation therapy?-a review of preclinical studies. Cancers (Basel). (2022) 14(15):3625. doi: 10.3390/cancers14153625, PMID: 35892895 PMC9330631

[10] Billena C Khan AJ . A current review of spatial fractionation: back to the future? Int J Radiat Oncol Biol Phys. (2019) 104:177–87. doi: 10.1016/j.ijrobp.2019.01.073, PMID: 30684666 PMC7443362

[11] Zhang H Wu X Zhang X Chang SX Megooni A Donnelly ED . Photon grid radiation therapy: A physics and dosimetry white paper from the radiosurgery society (Rss) grid/lattice, microbeam and flash radiotherapy working group. Radiat Res. (2020) 194:665–77. doi: 10.1667/RADE-20-00047.1, PMID: 33348375

[12] Yan W Khan MK Wu X Simone CB 2nd Fan J Gressen E . Spatially fractionated radiation therapy: history, present and the future. Clin Transl Radiat Oncol. (2020) 20:30–8. doi: 10.1016/j.ctro.2019.10.004, PMID: 31768424 PMC6872856

[13] Wu X Perez NC Zheng Y Li X Jiang L Amendola BE . The technical and clinical implementation of lattice radiation therapy (Lrt). Radiat Res. (2020) 194:737–46. doi: 10.1667/RADE-20-00066.1, PMID: 33064814

[14] Duriseti S Kavanaugh JA Szymanski J Huang Y Basarabescu F Chaudhuri A . Lite Sabr M1: A phase I trial of lattice stereotactic body radiotherapy for large tumors. Radiother Oncol. (2022) 167:317–22. doi: 10.1016/j.radonc.2021.11.023, PMID: 34875286

